# Mediastinal B-Cell Lymphoma Presenting with Jugular-Subclavian Deep Vein Thrombosis as the First Presentation

**DOI:** 10.1155/2015/929127

**Published:** 2015-03-04

**Authors:** Sherif Ali Eltawansy, Mana Rao, Sidney Ceniza, David Sharon

**Affiliations:** ^1^Department of Internal Medicine, Monmouth Medical Center, Long Branch, NJ 07740, USA; ^2^Department of Oncological Medicine, Monmouth Medical Center, Long Branch, NJ 07740, USA

## Abstract

Jugular venous thrombosis infrequently could be secondary to malignancy and has seldom been reported secondary to mediastinal large B-cell lymphomas. The postulated mechanisms are mechanical compression that leads to stagnation of blood in the venous system of the neck and/or an increase in the circulating thrombogenic elements that could cause venous thromboembolism as a paraneoplastic phenomenon. We report the case of a middle aged male presenting with right sided neck pain and arm swelling secondary to ipsilateral jugular-subclavian deep vein thrombosis. Investigations revealed it to be secondary to a mediastinal mass shown on CT scan of the chest.

## 1. Introduction

Malignancies have a causal association with deep vein thrombosis which can be secondary to mechanical pressure of the tumor on the venous system draining blood from the affected part of the body or it could be a systemic phenomenon secondary to thrombogenic material released into the circulation from the tumorigenic tissue. Venous thromboembolism (VTE) is found at autopsy in at least 50% of cancer patients [1, 2]. However, assessment of the true incidence of VTE in cancer patients is difficult because most of these patients receive chemotherapy or hormonal therapy, both of which can precipitate VTE [3]. In addition, many cancer patients have indwelling central venous lines, which can also initiate thrombotic events in relation to the catheter [4]. One of the most common causes of upper extremity vein thrombosis is prolonged central vein catheterization. About 66% of patients with internal jugular vein (IJV) catheters have proof of thrombus formation on ultrasound or at autopsy [5]. Spontaneous internal jugular vein thrombosis (IJVT) without a predisposing cause has been rarely reported [6]. However, IJVT may be secondary to malignancies [7, 8].

## 2. Case Presentation 

**Figure 1:**
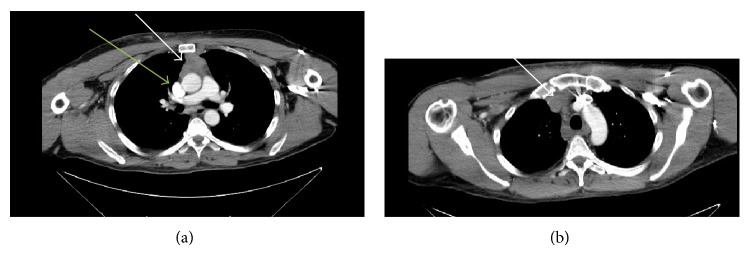
CT chest with contrast: 6/29/2014. Technique: contiguous transaxial images were obtained from the thoracic inlet to the upper abdomen with contrast. 125 cc omnipaque 350. (a) Thrombosis of the right internal jugular vein with extension to the right innominate vein and subclavian vein was noted (green arrow). The origin of the great vessels was patent. There was an anterior mediastinal mass (white arrow) present measuring 4.4 × 3.4 cm. Fibrotic changes were noted at the right base. There was no pulmonary mass or infiltrate. The thin cut after contrast series demonstrated no evidence of large pulmonary emboli. Fluid attenuation was noted just superior to the azygos arch likely representing prominence of the pericardial recess. There was a 2.8 × 1.1 cm lymph node in the subcarinal region image. There were prominent right axillary lymph nodes noted. (b) There was prominent soft tissue attenuation in the anterior mediastinum which was abutting the thrombosed jugular and innominate veins (white arrow). This was likely superior extension of the previously mentioned anterior mediastinal mass.

**Figure 2:**
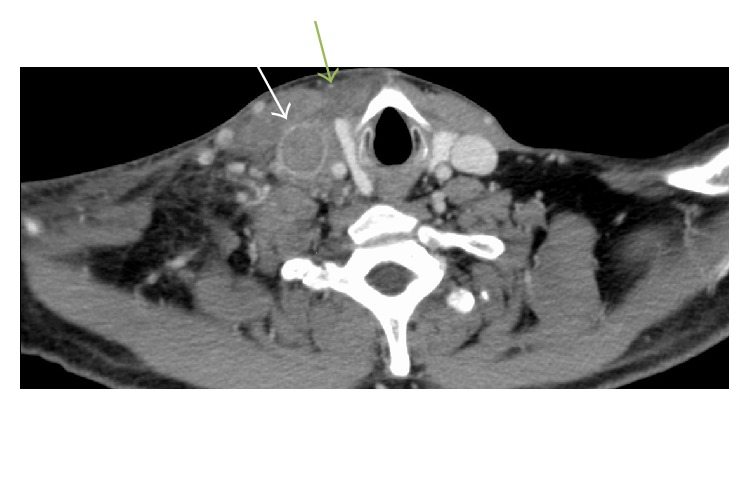
Thrombosis of the right internal jugular vein is noted (white arrow). There is hyperemia of the vein wall, and the vein is dilated to 2.9 × 2.4 cm. There is extension of the thrombus into the subclavian vein. There is extension of thrombus into the innominate vein as well. There is a mediastinal soft tissue density measuring 4.1 × 2.8 cm (green arrow). The right vertebral artery is diminutive in size. The basilar artery is patent and the vertebral arteries are patent. The left internal jugular vein is patent. The origins of great vessels are patent. The thrombus in the right jugular vein extends up to the level of the skull base. The extension of the thrombus into the sigmoid sinus on the right is to be noted. The dilated jugular vein is exerting mass effect on the adjacent thyroid gland.

**Figure 3:**
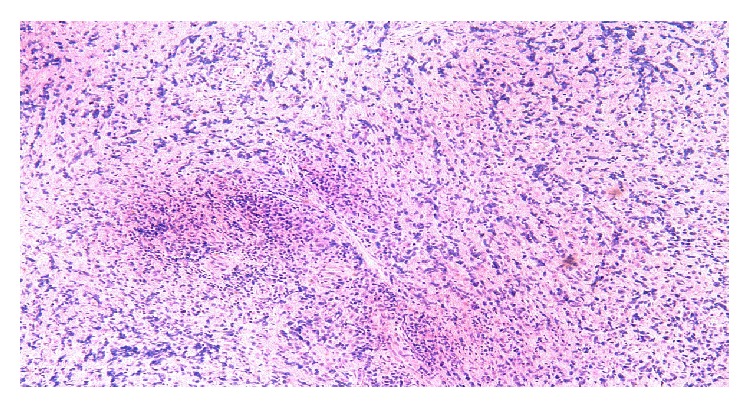
Large transformed lymphocytes are identified (low power). They are uniformly CD20+. Having in mind that there is a background sclerosis, the diagnosis is mediastinal large B-cell lymphoma with sclerosis. If the disease is localized to the mediastinum, the prognosis of this lymphoma is excellent. Stains used in this study: CD20, CD3, CD30, and CD10.

**Figure 4:**
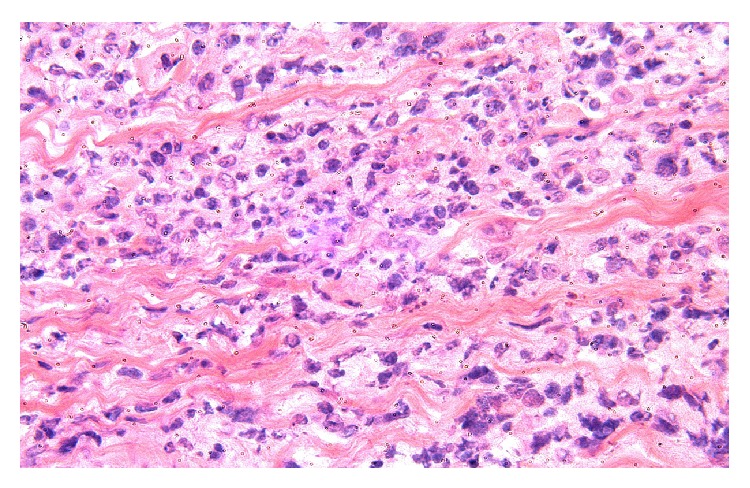
Mediastinal large B-cell lymphoma biopsy (high power).

**Figure 5:**
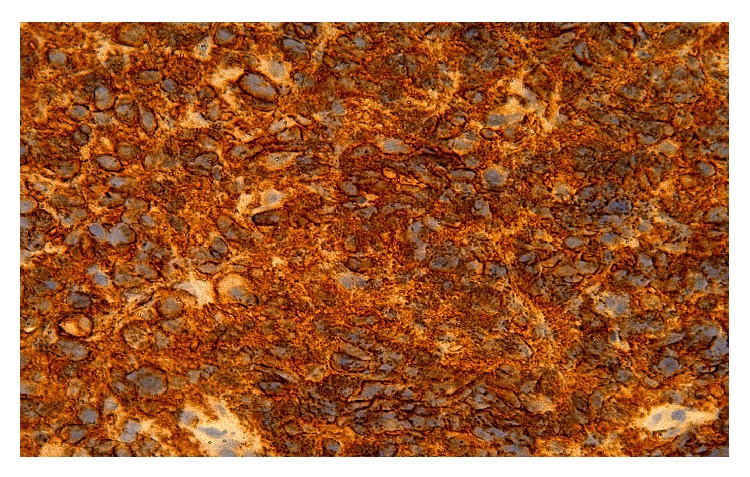
CD20 40x. Mediastinal large B-cell lymphoma biopsy CD20 40x.

**Figure 6:**
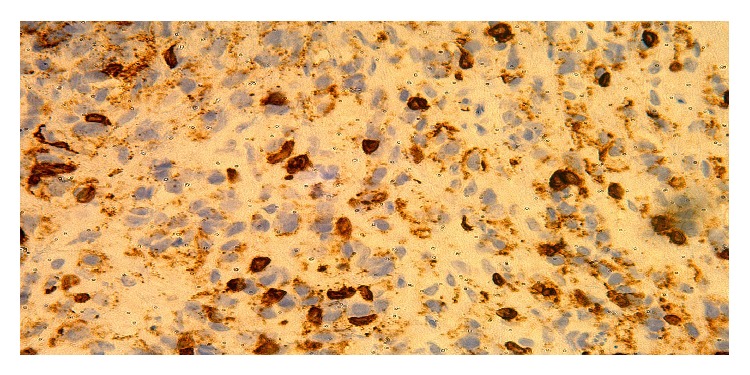
CD23 40x. Mediastinal large B-cell lymphoma biopsy CD23 40x.

A 51-year-old Caucasian male with no significant past medical history presented to the emergency room with a one-week history of right sided acute neck and arm pain associated with progressive swelling in the right upper extremity. He did not have such complaints in the past and had never seen a physician. He was smoking for 30 years (1 pack per year) but quit 5 years ago. His family history was significant only for coronary artery disease. Doppler ultrasound of the right upper extremity showed an occlusive thrombus in the right internal jugular vein; a nonocclusive thrombus in the right subclavian vein with minimal flow is within this vessel. The remaining veins including the axillary and brachial veins are patent. Hypercoagulable status work-up was unrevealing and meanwhile the patient was started on enoxaparin subcutaneously with a 1 mg/kg dose every 12 hours. Computed tomography (CT) scan of the chest and the neck with contrast showed a singular anterior mediastinal mass with persistent thrombosis of the right internal jugular, subclavian and innominate veins (Figures 1(a) and 1(b)). There was questionable extension of the clot into the right sigmoid sinus (Figure 2). CT brain and CT abdomen and pelvis were normal. Right video assisted thoracoscopic surgery was done and a biopsy of the anterior mediastinal mass was obtained with no complications. Biopsy showed mediastinal large B-cell lymphoma with sclerosis (Figures 3, 4, 5, and 6). Bone marrow biopsy was obtained and excluded the involvement of bone marrow. Cytogenetics study for lymphoma staging showed an apparently normal 46, XY male complement. No apparent numerical or structural abnormalities were evident. Flow cytometry study showed that the bone marrow was not involved by B-cell lymphoma morphologically and immunophenotypically. Patient started chemotherapy sessions and was continued on enoxaparin subcutaneously. The PET scan was done, showing two large hypermetabolic nodal masses of the mediastinum representing the lymphoma. One component of the lower mass extends into the anterior mediastinum. The patient started chemotherapy with R-CHOP-regimen.

## 3. Discussion

The internal jugular vein (IJV) drains blood from the transverse, sigmoid, and inferior petrosal sinuses of the brain and originates in the posterior compartment of the jugular foramen at the base of the skull. It also drains blood from the face and courses through the neck in proximity with the carotid artery, ultimately meeting the subclavian vein to form the brachiocephalic (innominate) vein.

Internal jugular vein thrombosis (IJVT) may be the result of various conditions of which the most common is maintenance of central venous access devices such as internal jugular vein catheters. The other known and reported reasons for such an occurrence are otolaryngological infections leading to Lemierre syndrome, skin and soft tissue infections around the neck, intravenous drug abuse (direct injection into the IJV), recent head/neck surgery, hypercoagulable states such as protein C or S deficiency, factor V Leiden, antithrombin III deficiency, polycythemia, and hyperhomocysteinemia. Malignant neck masses such as cancers originating in the thyroid or thymus may also precipitate this condition. Ovarian hyperstimulation has also been reported to cause IJVT [9].

The theory behind thrombus formation is the real time interpretation of an archaic concept proposed by Virchow: interruption of blood flow leading to venous stasis, irritation of the blood vessel or its vicinity described as endothelial injury, and phenomena of blood coagulation now known as hypercoagulability.

The way cancers cause venous thrombosis can be either one or both of these: anatomic compression of the blood vessel by tumor bulk or dissemination of prothrombotic material from the tumorigenic tissue (Trousseau's syndrome).

Kunimasa et al. reported a case of spontaneous left sided internal jugular vein thrombosis secondary to a left upper lobe nonsmall cell lung cancer [10]. Serinken et al. reported a case of IJVT secondary to a soft tissue infection in the vicinity of the IJV [11]. Ishida et al. reported a case of IJVT that possibly led to spontaneous spinal epidural hematoma [12]. Chlumský and Havlín encountered a case of IJVT without an obvious cause [13]. Ghatak et al. presented a case of spontaneous pulmonary embolism, IJVT, and subclavian vein thrombosis in a patient with septic shock who was found to have factor V Leiden mutation and activated protein C resistance and was curiously reported to have dengue IgM positive [14]. Lønnebakken et al. found an internal jugular vein thrombosis incidentally secondary to substernal goiter [15].

Another case with jugular vein thrombosis was reported secondary to a deep neck lipoma [16].

To date, there are no set guidelines for the treatment of internal jugular vein thrombosis. Sheikh et al. report the use of low molecular weight heparin followed by warfarin and superior vena cava filter to mitigate the possibility of clot expansion and extension which could ultimately lead to pulmonary embolism, in a select subset of patients who were not deemed to be candidates for anticoagulation [6]. Lymphoma is a well-known blood/lymphatic malignancy with different clinical presentations that may vary from the occurrence of B symptoms (fever, night sweats, and weight loss) to enlargement of lymph nodes and involvement of extranodal sites such as the skin, liver, gastrointestinal tract, and bone marrow. Less common presentations include but are not limited to symptoms from the primary tumor compressing its surrounding normal tissue. This has been reported in Burkitt's lymphoma causing abdominal pain and fullness, given its predilection for the abdomen. Albeit, it is infrequent to find that superior vena cava syndrome has been reported to occur with tumors that compress the great vessels in the neck and mediastinum [10].

SVC syndrome is even more common in patients with primary mediastinal large B-cell lymphoma with sclerosis, an unusual and aggressive NHL subtype that represents 3 to 7 percent of all diffuse large cell lymphomas. Patients typically present with a rapidly enlarging anterior mediastinal mass, frequently with associated SVC syndrome. In one report of 30 patients, 17 (57 percent) had SVC syndrome at presentation [17].

Our patient was eventually diagnosed with mediastinal large B-cell lymphoma with sclerosis. It is a seldom occurrence and hence as physicians it is imperative to be cognizant about the rare but certainly plausible presentation of mediastinal masses as IJVT and SVC syndrome. A high index of suspicion based on age, history, and appropriate clinical characteristics is vital.

## 4. Conclusion

It is imperative to be cognizant about the infrequent but certainly plausible presentation of mediastinal masses as jugular vein thrombosis and superior vena cava syndrome. The physician must maintain a high index of suspicion based on age, history, and clinical characteristics.

## 5. Learning Objective

Internal jugular vein thrombosis is a clinical finding confirmed on the ultrasound in patients presenting with symptoms like arm swelling and neck pain. Mostly it is secondary to clear causes like central venous line catheterization or intravenous drug abuse by history. If source is unidentified, work-up should be initiated to look for rare causes (like malignancy in our case) that may need prompt management.
